# The myth of aortic valve annulus changes in aortic valve disease

**DOI:** 10.3389/fcvm.2023.1302992

**Published:** 2023-12-14

**Authors:** Yanren Peng, Huijun Hu, Xiaorong Shu, Yongqing Lin, Weibin Huang, Shuwan Xu, Ruqiong Nie

**Affiliations:** ^1^ Department of Cardiovascular Medicine, Sun Yat-sen Memorial Hospital, Guangzhou, China; ^2^ Department of Radiology, Sun Yat-sen Memorial Hospital, Guangzhou, Guangdong, China

**Keywords:** aortic regurgitation, aortic stenosis, aortic annulus, computed tomography, transcatheter aortic valve replacement

## Abstract

**Background:**

The characteristics of aortic annulus changes in aortic regurgitation (AR) patients are poorly understood, and predictive factors among aortic valve disease are yet to be established.

**Objective:**

This study seeks to elucidate the pattern of annular size fluctuations across different cardiac phases in AR patients and to identify predictors for annular enlargement during either systole or diastole in aortic valve diseases.

**Methods:**

A retrospective analysis was conducted on 55 patients with severe aortic valve diseases, including 26 patients with aortic stenosis (AS) and 29 with AR, to discern the two groups' contrasting and analogous patterns of annular changes. The patient sample was expanded to 107 to investigate the factors influencing the size of the annulus during different cardiac phases. Based on our findings, patients were then divided into two groups: those with an annulus that is larger during systole (83 patients) and those where the annulus is larger during diastole (24 patients).

**Results:**

Typically, AR patients exhibit a dynamic annulus, with both perimeter and area being largest during mid-systole. These dimensions diminish progressively and then increase again in early diastole, a pattern consistent with observations in AS patients. Among 107 patients, 21% had diastolic enlargement. Systolic measurements would lead to prosthesis undersizing in 17% of these. Male gender and lower systolic annulus minimum relative to body surface area (AnMin index) were predictors of diastolic enlargement, with ROC curve areas of 0.70 and 0.87 for AR and AS, respectively.

**Conclusions:**

Systolic measurements are recommended for AR patients. Gender and the AnMin index are significant predictors, particularly potent in AS patients.

## 1. Introduction

Transcatheter aortic valve replacement (TAVR) has become a pivotal treatment for patients with severe aortic valve diseases, where accurate annular sizing is crucial for choosing the appropriate valve prosthesis (1). Computed tomography (CT) is preferred for this purpose due to its precision (2). Accurate measurement of the annulus is essential in TAVR planning, as selecting a smaller-than-optimal valve size can lead to complications such as paravalvular leakage and device dislodgment (3). Existing research has expanded TAVR applications in AR, resulting in promising outcomes (4). However, AR patients often lack valvular calcification, reducing anchoring strength and increasing the risk of paravalvular leakage and device dislodgment compared to AS patients (5, 6).

It is widely recognized that conformational changes during systole frequently lead to increased annular area and perimeter compared to diastole (7). Although systolic measurements are conventionally used for sizing, diastolic data are crucial in assessing aortic valve morphology, particularly in AR patients relying on annular anchoring without leaflet calcification. Understanding the annular changes in AR patients and identifying the phase with the largest annulus is therefore critical. However, the annular variations in AR patients remain inadequately investigated (8). Conversely, the largest annular dimensions may manifest during diastole due to inversed dynamism in septal hypertrophy (2, 9). Nonetheless, there remains uncertainty about the role of septal hypertrophy and other factors in predicting whether the annulus is larger in systole or diastole. Understanding these predictive indicators may be helpful in determining the phase with the largest annulus (systole/diastole) for valve sizing, potentially reducing the risk of complications like paravalvular leakage and improving long-term patient outcomes (10, 11).

Hence, this study pursues two primary objectives: to elucidate the cyclic variations in patients with aortic regurgitation and to identify the predictors of annular enlargement during systole or diastole. Through these aims, we aim to contribute to refining TAVR strategies and enhancing patient outcomes in both AS and AR cohorts.

## 2. Methods

### 2.1. Patient cohort and grouping

Our study initially involved a consecutive cohort of 58 patients diagnosed with severe symptomatic aortic disease, referred for transcatheter aortic valve implantation (TAVI), and who underwent preprocedural ECG-gated cardiac computed tomography angiography (CTA) at our institution from March 2022 to March 2023. This phase focused on examining annular dimension changes at 10% intervals across the cardiac cycle, particularly between the 20%-80% phase range (Table 1). Of these patients, 55 were eligible for inclusion, with 26 classified in the AS group and 29 in the AR group, after excluding those with prior aortic valve replacement history.

**Table 1:** Baseline characteristics of the non-expanded cohort.

	Patients With Aortic Stenosis	Patients With Aortic Regurgitation
	(*n* = 26)	(*n* = 29)
Age, yrs	69.8 ± 8.6	68.0 ± 10.0
Female	12 (46)	6 (21%)
BMI, kg/m^2^	23.8 ± 3.9	23.2 ± 3.4
BSA, m^2^	1.60 ± 0.18	1.69 ± 0.17
LVEDD, mm	53 ± 8	64 ± 8
LVEF, %	59 ± 13	55 ± 13
IVS, mm	12 (11, 14)	11 (9, 12)
BAV	14 (56)	2 (7)
Type0	8 (31)	1 (3)
Type1	4 (15)	1 (3)
Type2	2 (9)	0(0)

BAV, bicuspid aortic valve; BMI, body mass index; BSA, body surface area; IVS, interventricular septum; LVEDD, left ventricular end-diastolic dimension; LVEF, left ventricular ejection fraction;.

Recognizing the need for a broader data scope, the study's scope was extended to include 112 cases from March 2021 to March 2023. This expansion aimed to identify predictive factors for whether the annulus was larger during systole or diastole. After similar exclusion criteria, 107 patients were incorporated into this extended segment. This cohort included patients from the initial period, thus overlapping the two timeframes. The analysis in this phase utilized strategically selected imaging phases for optimal image quality, assessing the annulus predominantly between 20%–35% for systolic and 65%–80% for diastolic phases. The patients were then categorized into systolic-enlarged (*n* = 84) and diastolic-enlarged (*n* = 23) groups, as detailed in Table 2.

**Table 2:** The baseline of the expanded cohort.

	Patient with systolic annulus enlargement	Patient with diastolic annulus enlargement	*P* value
	(*n* = 84)	(*n* = 23)	
Age, yrs	69.8 ± 7.3	68.4 ± 6.5	0.401
Female, %	37 (44)	3 (13)	0.007
BMI, kg/m^2^	23.4 ± 3.9	23.1 ± 4.3	0.754
BSA, m^2^	1.62 ± 0.19	1.70 ± 0.12	0.016
Cardiac risk factors			
HTN, %	53 (63)	12 (52)	0.478
DM, %	19 (23)	5 (21)	1
CAD, %	35 (42)	11 (48)	0.771
Electrocardiogram			
AF, %	13 (15)	4 (17)	0.758
AVB, %	11 (13)	4 (17)	0.735
BBB, %	9 (11)	2 (9)	1
Echocardiography			
LVEDD, mm	59 (51, 64)	57 (52, 61.5)	0.492
LVEF, %	60 (50, 65)	60 (46.5, 65.5)	0.979
IVS, mm	12 (10, 13)	12 (10, 14)	0.421
IVSH radio, %	43 (51)	14 (61)	0.556
Severe AS, %	44 (52)	13 (57)	0.907
>=3 AR, %	49 (58)	12 (52)	0.771
>=3 MR, %	12 (14)	4 (17)	0.968
Structural parameters			
Calcification volumn	65.5 (0, 702.2)	170.6 (0, 603)	0.863
Annulus calcification, %	29 (35)	7 (30)	0.906
LeafThickness, %	50 (60)	16 (70)	0.525
BAV, %	25 (30)	7 (30)	1
Type 0	15 (18)	4 (17)	
Type 1	8 (10)	3 (13)	
Type 2	2 (2)	0	
Systolic annulus perimeter, mm	80.4 ± 8.4	79.3 ± 8.0	0.569
Systolic annulus Area, mm^2^	504.8 ± 104.9	484.5 ± 94.4	0.378
Systolic annulus minimum, mm	22.8 ± 2.4	21.6 ± 2.3	0.046
Systolic annulus maximun, mm	28.1 ± 3.2	28.1 ± 3.2	0.978
Systolic annulus EIicity	0.19 ± 0.06	0.23 ± 0.07	0.010
Systolic LVOT minimun, mm	22.1 ± 3.6	21.1 ± 3.6	0.234
Systolic LVOT maximun, mm	29.5 ± 4.0	29.4 ± 3.7	0.870
Systolic LVOT average, mm	25.8 ± 3.6	25.2 ± 3.3	0.465
Systolic LVOT EIicity	0.25 ± 0.07	0.28 ± 0.09	0.124
Systolic LVOT/Annulus	1.03 ± 0.14	1.03 ± 0.14	0.966

AF, Atrial fibrillation; AVB, Atrioventricular Block; AS, aortic stenosis; AR, aortic regurgitation; BBB, Bundle Branch Block; BMI, body mass index; BSA, body surface area; CAD, coronary artery disease; DM, diabetes mellitus; HTN, hypertension; IVS, interventricular septal; IVSH, interventricular septal hypertrophy; LVEDD, left ventricular end-diastolic dimension; LVOT, left ventricular outflow tract; MR, Mitral Regurgitation.

### 2.2. Scan protocol

Contrast-enhanced multi-detector computed tomography (MDCT) examinations were performed using a third-generation dual-source CT system (Siemens Somatom Definition Flash; Siemens Healthcare, Erlangen, Germany) with a collimation of 192 × 0.6 mm, fixed tube potential of 70–120 kV, and Automated real-time anatomical tube current modulation (Care Dose 4D; Siemens). Non-ionic iodine contrasts medium iopamidol (370 mg/ml Iopamiro, Shanghai Bracco Sine Pharmaceutical Corp Ltd) was used. The total volume of the contrast agent was calculated as injection rate × (delay time + scan time), followed by a 40 ml injection of 0.9% saline solution at a flow rate of 5 ml/s after the completion of the iodine contrast agent injection. The scanning range was from 1 cm below the tracheal carina to the cardiac diaphragm. The prospective electrocardiogram (ECG)-gated step-and-shoot technique (SAS) was used with an advanced model iterative reconstruction algorithm (ADMIRE, intensity 2) for image reconstruction at a slice thickness of 0.6 mm and a reconstruction increment of 0.4 mm with convolution kernel Bv36. For the 55-patient cohort from March 2022 to March 2023, we reconstructed the contrast-enhanced scan's 3-dimensional dataset at 10% intervals throughout the cardiac cycle, resulting in a comprehensive 20%–80% CT dataset for aortic annulus assessment. In the extended 107-patient cohort from March 2021 to March 2023, we strategically selected the 20%–35% phase for systolic analysis and the 65%–80% phase for diastolic analysis to ensure optimal image quality for precise annular evaluation. Definitions of additional clinical parameters can be found in Supplementary Table S2.

### 2.3. Assessment of the aortic annulus

Two independent researchers (2 and 4 years) analyzed the complete dataset using 3mensio software (Pie Medical, The Netherlands). Inconsistencies were resolved by measuring again. The aortic annulus was defined as the virtual circumferential connection of the aortic leaflets' basal attachments (virtual basal ring). For cases of bicuspid aortic stenosis, the aortic valve annulus was determined by initially identifying the nadirs of the two sinuses and then identifying the plane with the smallest cross-sectional area within the planes defined by these nadirs as the annulus plane. Several measurements were taken at each phase, including the minimum and maximum diameters of the aortic annulus and its cross-sectional area and perimeter. The ellipticity index (EI) was calculated using the formula: 1—(maximum to minimum) × 100% (12).

### 2.4. Statistical analysis

All statistical analyses were conducted using R language (version 4.1.2). The normality of all measured variables was confirmed using the Shapiro-Wilk test. Continuous variables were presented as mean ± standard deviation and compared using Student's *t*-test when they followed a normal distribution. The median and interquartile range were reported for non-normally distributed variables and compared using the Mann-Whitney *U*-test. Categorical data were compared using the *χ*^2^ test or Fisher's exact test. To account for the potential confounding effect of body size variations, diameter, area, and perimeter measurements were indexed for body surface area. Correlation analysis was performed using Spearman's method. Univariate and multivariate logistic regression analyses were conducted to explore the variables associated with an expanded aortic annulus in both systolic and diastolic phases, determining the phase of greater annular expansion. The predictive performance was assessed using receiver operating characteristic curves (ROC) and the area under the ROC curve (AUC). *P* < 0.05 was considered statistically significant. The Bland and Altman method tested Interobserver variability in 36 randomly selected study subjects. In addition, the intraclass correlation coefficient was determined.

## 3. Results

### 3.1. Variation of annular dimensions

As demonstrated in Table 3 and Supplementary Figure S1, the variation in annular dimensions across the cardiac cycle in patients with AR resembles that in patients with AS. Specifically, the annular diameter progressively enlarges from mid-systole to early diastole yet gradually diminishes during mid-diastole. Notably, in the case of AR, 86% (25/29) of the patients exhibited a larger annular dimension during systole. Likewise, in AS patients, 85% (22/26) demonstrated a larger systolic annular diameter. As illustrated in Supplementary Figure S2, paired *t*-tests revealed significant differences between the systolic and diastolic annular dimensions regarding both annular circumference and area. Interestingly, no significant differences were observed between the 20% and 30% phases or the 70% and 80% phases of the cardiac cycle. Intriguingly, the annular dimensions at the 50% phase also showed no significant differences compared to the 70% and 80% phases.

**Table 3:** Variation of annular Dimensions in patients with aortic stenosis or regurgitation.

	Patients With Aortic Stenosis				Patients With Aortic Regurgitation			
	(*n* = 26)				(*n* = 29)			
	Perimeter	Area	Minimun	Elicity	Perimeter	Area	Mininum	Elicity
20%	78.4 ± 7.5	480.1 ± 92.9	22.3 ± 2.3	0.18 ± 0.07	85.0 ± 8.1	567.3 ± 121.4	24.1 ± 2.1	0.18 ± 0.06
30%	78.3 ± 7.8	477.2 ± 94.5	22.0 ± 2.3	0.19 ± 0.07	84.6 ± 8.7	562.0 ± 127.6	23.6 ± 2.3	0.20 ± 0.05
40%	77.9 ± 7.9	470.3 ± 96.3	21.6 ± 2.4	0.21 ± 0.06	83.3 ± 9.3	543.9 ± 136.6	23.1 ± 2.8	0.21 ± 0.07
50%	76.6 ± 8.0	451.4 ± 96.4	20.8 ± 2.5	0.24 ± 0.07	81.6 ± 8.7	518.3 ± 127.7	22.6 ± 2.6	0.22 ± 0.08
60%	76.1 ± 7.9	443.9 ± 90.1	20.5 ± 2.2	0.24 ± 0.06	81.0 ± 8.8	506.4 ± 127.4	21.8 ± 3.0	0.24 ± 0.08
70%	76.4 ± 7.9	450.5 ± 93.8	20.8 ± 2.3	0.23 ± 0.07	82.0 ± 9.0	519.8 ± 129.3	22.3 ± 2.9	0.23 ± 0.08
80%	76.2 ± 8.1	447.7 ± 94.6	20.7 ± 2.3	0.23 ± 0.06	82.3 ± 8.9	525.2 ± 129.0	22.2 ± 2.7	0.24 ± 0.07

### 3.2. Impact of annular variation on prosthetic valve selection

**Figure 1:**
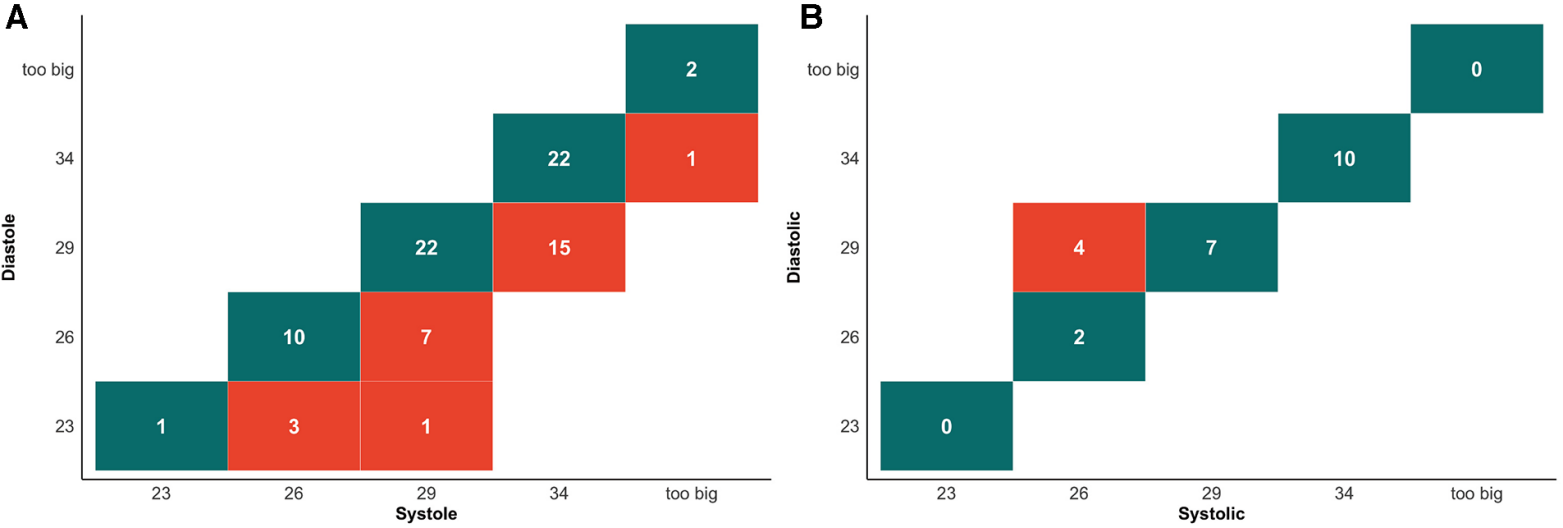
Cross tabulation of valve selection between systolic and diastolic measurements for perimeter. (**A**) Cross tabulation of valve selection in patient with systolic enlargement group. Values highlighted in dark green boxes indicate concordance between valve selection in diastolic and systolic measurements. Values highlighted in light red show the number of cases where systolic measurements would result in a larger valve size recommendation. (**B**) Cross tabulation of valve selection in patient with systolic enlargement group. Values highlighted in dark green boxes indicate concordance between valve selection in diastolic and systolic measurements. Values highlighted in light red show the number of cases where diastolic measurements would result in a larger valve size recommendation.

Our study stratified patients into two groups based on whether the annular dimensions were larger during systole or diastole. Out of 107 patients, 84 exhibited larger annular dimensions during systole, accounting for 79%, whereas 23 had larger dimensions during diastole, making up 21% of the sample. According to the criteria in Supplementary Table S3, prosthetic valve sizes were selected based on systolic or diastolic annular dimensions sourced from the annular circumference. Within the group where systolic measurements were larger, 32% (27/84) would have been sized down one valve size due to diastolic measurements. Conversely, in the group with larger diastolic dimensions, 17% (4/23) would be sized down one valve size due to systolic measurements (Figure 1).

### 3.3. Logistic regression analysis to predict factors influencing larger annular dimensions in systole and diastole

**Figure 2:**
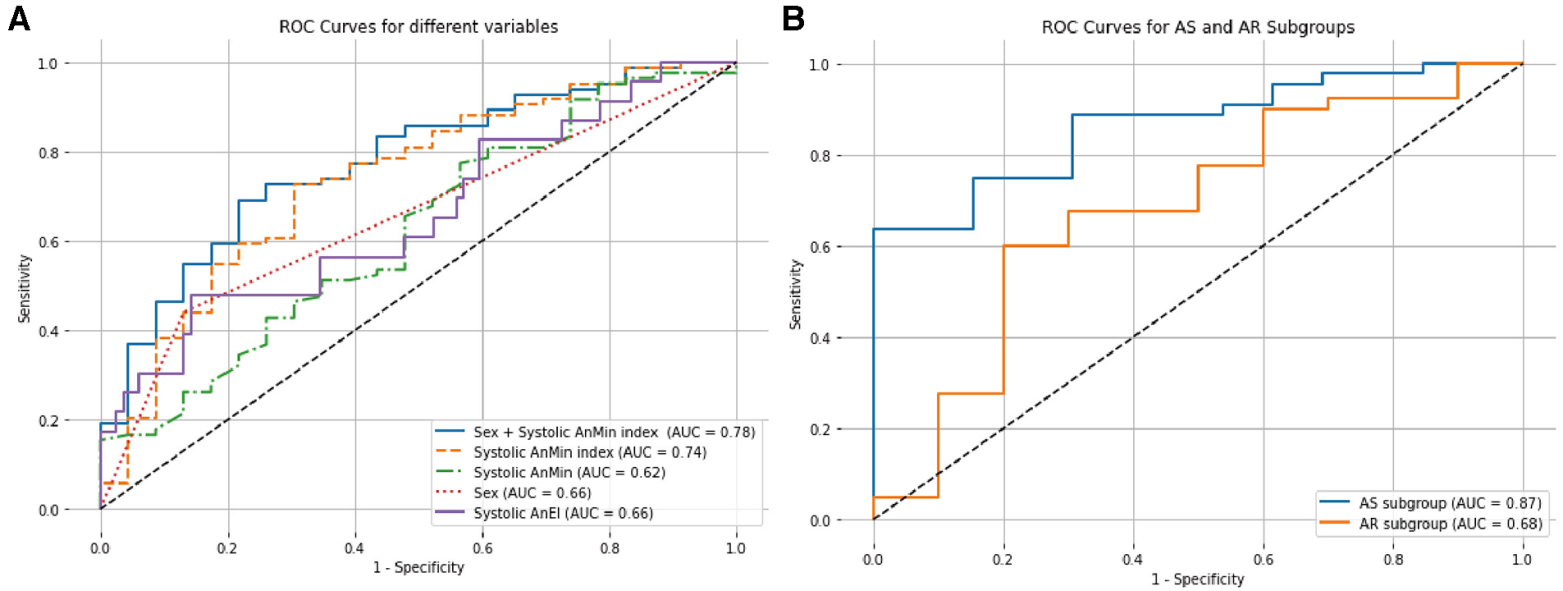
ROC curves for factors influencing annular dimensions in systolic and diastolic phases. (**A**) The figure displays the ROC curves for various predictors in determining whether the aortic annulus is larger during systole or diastole. The solid blue line illustrates the combined predictive power of sex and the systolic AnMin index. The dashed orange line represents the Systolic AnMin index, the solid purple line for Systolic AnEI, the dotted red line for Sex, and the dashed green line for Systolic AnMin. (**B**) The figure compares the ROC curves for the AS and AR subgroups based on the combined sex and Systolic AnMin index. The solid blue line represents the AS subgroup, and the solid orange line corresponds to the AR subgroup. AUC, areas under the curve; ROC, receiver operating characteristic.

In our univariate logistic regression analysis (Table 4), we identified gender and the systolic AnMin index as significant factors in determining the phase of annular enlargement in patients. Specifically, the analysis indicated that males are more likely to be classified into the diastolic enlargement group, as shown by a negative coefficient for gender. This suggests that males, compared to females, have a lower probability of having a larger annulus during systole. In contrast, a positive coefficient for the systolic AnMin index implies that a higher index is associated with an increased likelihood of being in the systolic enlargement group. Due to collinearity between systolic AnEI and the AnMin index, we included only gender and the latter in our multivariable logistic regression model. Both factors were found to be significant, as detailed in Table 5. This multivariate analysis substantiates the importance of these factors in predicting annular dimensions during different cardiac phases. ROC curve analysis revealed a high predictive value for the model incorporating gender and the systolic AnMin index, with an AUC of 0.78. Using the maximum Youden index, the model demonstrated a sensitivity of 62%, a specificity of 83%, a positive predictive value of 93%, and a negative predictive value of 37% (Figure 2A). The diagnostic cut-off points for the systolic AnMin index were 14.4 mm/m^2^ for males and 11.6 mm/m^2^ for females.

**Table 4:** Univariate regression analysis of factors predicting whether the annulus is larger in systolic or diastolic phases.

Varaibles	β	OR	*P* value	CI
Age	0.029	1.030	0.396	0.962–1.102
Sex	−1.658	0.191	0.012	−3.156–−0.495
BSA	−2.640	0.071	0.059	−5.519–0.016
BMI	0.020	1.021	0.737	−0.096–0.143
LVOT/Annulus	−0.075	0.928	0.965	−3.391–3.316
Calcification volumn	0.000	1.000	0.821	0.999–1.001
IVS	−0.052	0.940	0.591	0.240–0.144
Basal IVS	0.023	1.024	0.788	−0.144–0.198
Systolic AnMin	0.205	1.228	0.049	0.007–0.420
Systolic AnMin index	0.552	1.735	0.001	0.237–0.916
Systolic AnEI	−12.204	<0.001	0.007	−21.766–−3.853

AnEI, annular eccentricity; AnMin, annulus minimum; AnMin index, annulus minimum relative to body surface area; BMI, body mass index; CI, Confidence Interval; IVSH, interventricular septal hypertrophy; OR, Odds Ratio.

**Table 5:** Multiple logistic regression for factors predicting whether the Annulus is larger in systolic or diastolic phases.

Varaibles	β	CI	*P* value	OR
Sex	−1.500	−2.919–−0.068	0.028	0.260
Systolic AnMin index	0.532	0.104–0.882	0.003	1.595

AnMin index, annulus minimum relative to body surface area; CI, Confidence Interval; OR, Odds Ratio.

### 3.4. Predictive efficacy in the AS and AR subgroups

We further investigated the predictive efficacy of gender and the systolic AnMin index within AS and AR subgroups. In the AS subgroup, gender and the systolic AnMin index effectively differentiated between patients with larger dimensions during systole vs. diastole, achieving an AUC of 0.87. Using the maximum Youden index, the model achieved a sensitivity of 63%, specificity of 100%, positive predictive value of 100%, and negative predictive value of 46%. The diagnostic cut-off points of the systolic AnMin index were 14.5 mm/m^2^ for males and 11.6 mm/m^2^ for females. For the AR subgroup, the AUC was 0.70, with a sensitivity of 69%, specificity of 67%, positive predictive value of 89%, and negative predictive value of 35% (Figure 2B). The diagnostic cut-off points of the systolic AnMin index were 13.9 mm/m^2^ for males and 12.0 mm/m^2^ for females. Interobserver variability and 95% limits were as follows: For the AnMin, −0.04 ± 0.65 mm (−1.32, 1.25); for the AnEI, 0.02 ± 0.07 (– 0.11, 0.16); for annulus area, 0.78 ± 12.98 mm^2^ (–24.65, 26.22), and annulus perimeter, 0.16 ± 0.96 mm (– 1.72, 2.05). The intraclass correlation coefficient was 0.96 for the AnEI and 0.99 for the AnMin, annulus area, and annulus perimeter.

## 4. Discussion

Our study reveals that most AR patients exhibit their largest annular dimensions during systole, both in terms of circumference and area, while a minority have larger annular sizes during diastole. Moreover, we expanded the cohort to identify predictive factors for whether the annulus is larger during systole or diastole. We found that gender and the systolic AnMin index effectively distinguished between the two. The predictive efficacy was strong in the AS subgroup but weaker in the AR subgroup. Clinically, for aortic diseases, measuring the annulus during systole is appropriate. Our study suggests that the need for additional diastolic measurements can be determined based on gender and the systolic AnMin index, which may potentially reduce the risk of undersizing the prosthetic valve due to larger diastolic annular dimensions.

In our study population, 86% of AR patients exhibited maximum annular dimensions during systole. The trend in annular dimension changes from systole to diastole in AR patients mirrored those observed in AS patients in other studies (13–16). This trend also held in normal individuals (17–19), and those with bicuspid valve malformations (20). Specifically, the annulus in AR patients was largest in mid-systole, decreased gradually to its smallest dimension in early diastole, and then increased again (Supplementary Figure S1). In concordance with our observations, Zhu et al. investigated a cohort of six high-risk patients diagnosed with severe, pure aortic regurgitation and undergoing TAVR. Their data underscored that the maximum annular circumference during mid-systole was significantly larger than its minimum value during mid-diastole (8).

**Figure 3:**
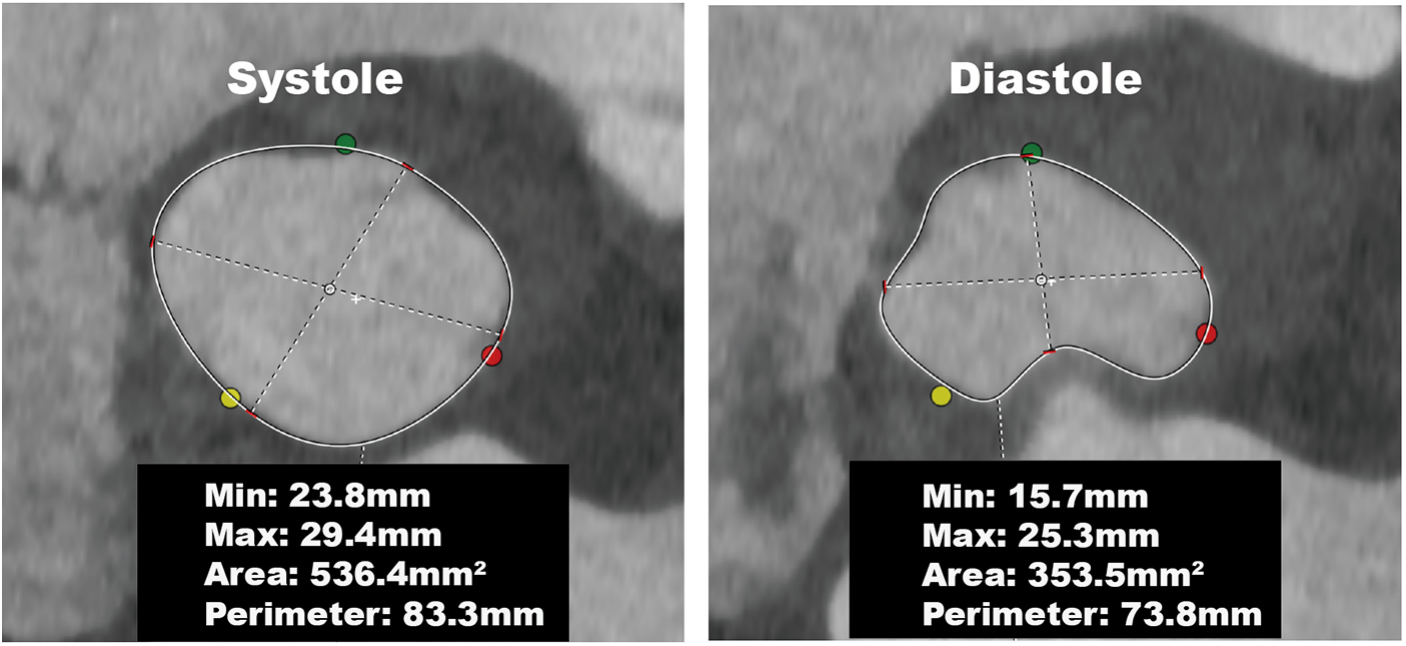
Annular changes in a patient with aortic regurgitation. The annulus was elliptical during systole and assumed a cloverleaf pattern during diastole, resulting in a significant reduction in the annular perimeter and area.

**Figure 4:**
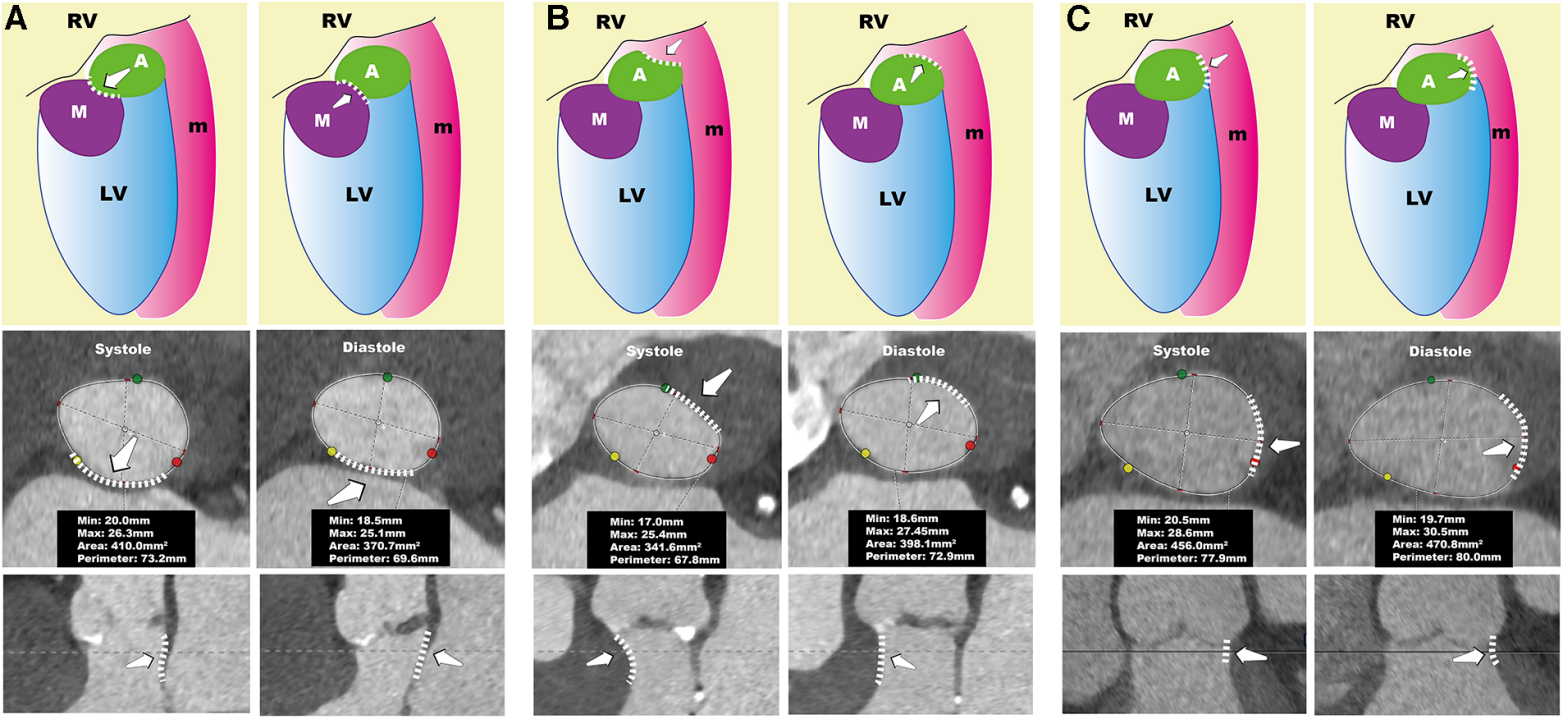
Systolic and diastolic conformation of the annulus. The left column of (**A–C**) represents the systolic phase, while the right column represents the diastolic phase. The top row shows schematic diagrams, the middle row presents the annular plane views, and the bottom row depicts longitudinal sectional views of the AnMin. In (**A**), during systole, the fibromuscular junction protrudes toward the left atrium, leading to a smaller AnMin, causing the annulus to appear larger in systole. In (**B**), during systole, the thickened interventricular septum contracts, leading to compression of the annulus, which reduces the perimeter and area of the annulus, making it larger in diastole. In (**C**), during diastole, the AnMax increases, which increases the perimeter and area of the annulus, making it larger in diastole. A, aortic valve; AnMax, annulus maximum; AnMin, annulus minimum;LV, left ventricle; M, mitral valve; m, muscle; RV, right ventricle.

Furthermore, in our tiny subset of AR patients (6%, 3/49), this difference in annular dimensions was even more pronounced—with a circumference greater than 6.2 mm. These patients displayed notable morphological changes; the annulus was elliptical during systole and assumed a cloverleaf pattern during diastole (Figure 3). Apart from patients with remarkable annular variation, most demonstrated changes similar to those seen in patients with AS. These alterations can be attributed to the forward movement of the fibromuscular junction between the left ventricle and the aorta (13). On CT, the annular plane is a virtual construct consisting of nearly 50% muscular tissue and the remaining fibrous tissue (21, 22). The fibrous tissue undergoes passive movement influenced by the pressure differential between the left ventricle and the left atrium. During systole, the fibromuscular junction protrudes toward the left atrium when left ventricular pressure exceeds left atrial pressure. Conversely, during diastole, when the ventricular pressure is lower than the atrial pressure, this fibromuscular junction bulges towards the left ventricle (Figure 4A, Supplementary Video S1).

To enhance the robustness of our findings, we strategically selected to measure the annular diameter at 20%–35% of the systolic phase and 65%–80% of the diastolic phase. This decision was based not only on the superior imaging quality available during these cardiac phases but also because the annular diameter demonstrated significant differences during these intervals (Supplementary Figure S2). We categorized patients into groups with larger annulus during systole or diastole based on these measurements. Subsequent analyses were conducted to compare these groups' general clinical data, electrocardiographic information, echocardiographic parameters, and aortic root structural metrics. Because it is now a widely accepted consensus among experts that the aortic annulus is generally larger during systole and sizing based on diastolic measurements holds the potential for unintended undersizing (2). Consequently, we restricted our predictive analysis to parameters collected during systole to optimize its applicability in a clinical setting.

We found significant differences in gender, body surface area, systolic AnMin, and systolic AnEI between the systolic-enlarged and diastolic-enlarged groups. Given the correlation between body surface area and the systolic AnMin (correlation coefficient *ρ* = 0.36, *P *< 0.01), we introduced a new variable, the ratio of the minimum annular diameter to body surface area, specifically termed the systolic AnMin index, for subsequent analyses. Subsequently, we conducted a univariate logistic regression analysis with the variables above. We found that gender, systolic AnMin, systolic AnMin index, and systolic AnEI are predictive factors for categorizing annular size. Due to the collinearity between the systolic AnMin index and the systolic AnEI (correlation coefficient *ρ* = −0.42, *P* < 0.01), and the higher predictive value of the former (AUC = 0.74 vs. AUC = 0.66), we opted for a multivariate logistic regression using gender and systolic AnMin index. Combining these two factors yielded better predictive accuracy for categorization (AUC = 0.78), with a sensitivity of 63% and a specificity of 83%. The rationale for selecting gender and the systolic AnMin index as parameters is based on their respective influences on AnMin. While AnMin shows a correlation with gender, the systolic AnMin index, calculated as AnMin divided by BSA, is used to standardize AnMin across varying body sizes. This standardization helps to mitigate the direct influence of body size, which may differ significantly between genders, allowing for a more accurate assessment of AnMin's independent effects in our multivariate analysis.

In individuals whose annular dimensions are larger during diastole, systolic thickening of the septal muscle tissue, especially in the basal segment of the interventricular septum, leads to compression of the annulus and a reduction in AnMin, as illustrated in Figure 4B. Consequently, a smaller AnMin during systole can be a distinctive marker for this subgroup. It is crucial to recognize the significant role of the basal segment of the interventricular septum in affecting the annulus since it forms the annulus's muscular component. In current clinical echocardiography practice, while the focus is often on the mid-segment of the interventricular septum, accurate measurement of the basal segment is also essential. Interventional cardiologists should be cognizant of how thickening in the basal segment can influence the annulus.

Our study shows that gender and the systolic AnMin index are effective predictors in the AS subgroup, with a high AUC of 0.87, sensitivity of 0.63, and perfect specificity. This effectiveness is less pronounced in the AR subgroup. In AS patients, a larger diastolic annulus is often due to septal muscle activity during the cardiac cycle, making the AnMin a good differentiator. However, in AR patients, diastolic annular enlargement can be caused by septal muscle expansion, an increase in left ventricular cavity size, or both, which complicates prediction (Figure 4C, Supplementary Video S2). We observed that AR patients with diastolic enlargement tend to have a smaller LVEDD, while those with systolic enlargement have a larger LVEDD (Supplementary Table S1). In cases of severe left ventricular dilation, annular size doesn't vary much between diastole and systole. This might be because patients with smaller LVEDD maintain diastolic function, leading to a larger annular diameter due to blood inflow during diastole. In contrast, a larger LVEDD often indicates reduced diastolic function, resulting in a larger annulus during systole. This could explain why severely AR affected hearts typically have a larger annulus during systole, as the condition is associated with an increased LVEDD, diminishing the impact of diastolic blood flow on the annulus's maximum diameter.

The choice of annulus has a significant impact on outcomes (23). Willson et al. compared the differences between CT and transesophageal echocardiography (TEE) in valve size selection and found that if CT recommended a larger size and TEE a smaller one, the incidence of paravalvular leak was 25% (3). In contrast, Steffen et al. found that although there were no differences in 30-day clinical outcomes, 3-year mortality rates were higher in the diastolic group [Society of Thoracic Surgeons score adjusted hazard ratio, 1.25 (1.07–1.46), *p* < 0.01] (11). These findings support the choice of the most considerable annulus size. Our results indicate that to identify the largest annulus, as many as 25% (27/107) should opt for systolic measurements, 4% (4/107) for diastolic measurements, and the remaining 71% (76/107) have consistent valve size choices (Figure 1). This contrasts with the findings of Murphy et al., who reported that 47% (237/507) required systolic measurements, 1% (3/507) needed diastolic measurements, and 53% (269/507) had consistent valve size choices (24). The discrepancies could be because Murphy et al.'s study involved a multi-center AS population, with Edwards Sapien 3 THV as the basis for valve size selection and a finer division of the “gray zone” for annulus size. In contrast, our study included AR patients and used Evolut PRO + for valve size selection.

Compared to CT and echocardiography, Cardiac Magnetic Resonance (CMR) has been shown to possess the highest accuracy and the least variability against the aortic annulus (25). Clinically, CMR can also be utilized for preoperative planning of TAVR, but its use is less common than CT due to the longer scanning times required and the need for patients to control their breathing. However, the advantages of CMR, such as the absence of radiation and higher tissue contrast, are significant. Existing studies have demonstrated that CMR assessments of aortic annulus movements and contractions are consistent with those obtained from CT (26). Scholars have utilized global longitudinal strain to evaluate changes in the muscular AV annulus and the fibrous AV annulus, finding that the direction of muscular annular deformation in patients with AR is opposite to that in patients with normal aortic valves (27). Therefore, by leveraging the high tissue contrast and the more detailed and accurate functional information provided by CMR, we can further analyze the detailed tissue changes of the aortic annulus to elucidate valve ring selection.

Our study demonstrates that it is reasonable and available for patients with AR to choose the systolic annulus as the measurement basis typically. This selection allows us to infer a general trend in the population at large: the annulus is largest during mid-systole, then rapidly diminishes to its smallest size during early diastole before gradually enlarging again. This trend has already been confirmed in patients with AS, in normal individuals, and in those with bicuspid valves; thus, we hypothesize it to be a general pattern. Combining gender and the systolic AnMin index can predict whether the annulus is larger during systole or diastole, with higher efficacy in patients with AS. Therefore, we could employ a simple calculation in clinical settings to determine if additional measurement during diastole is required. For patients falling below the diagnostic cut-off point, performing other diastolic annular measurements would be prudent to avoid the selection of an undersized prosthetic valve. Currently, our research serves as a preliminary exploration of the potential roles of study parameters such as AnEI and AnMin, alongside conventional parameters, within the scope of TAVR procedures. However, it remains to be conclusively determined whether these parameters can reliably predict paravalvular leakage during follow-up, and if their predictive value differs between AR and AS patient subgroups. All these aspects require further investigation.

## 5. Study limitation

In our study, we opted for prospective ECG-gated scanning instead of retrospective ECG-gated scanning to minimize radiation exposure. This method limited our capacity to capture all CT phases but adequately captured annular changes from mid-systole to mid-diastole, representing the general trend. We expanded our patient sample but restricted our measurements to the 20%–35% and 65%–80% phases to identify factors that could predict whether the annulus is larger in systole or diastole. Previous research has confirmed significant differences in these phases, adequately representing systolic and diastolic states. Finally, we determined that gender and the systolic AnMin index effectively predict whether the annulus is larger during systole or diastole. While the overall AUC was moderate and sensitivity was relatively low, these variables demonstrated high specificity, particularly in AS patients. Therefore, their application is especially recommended in patients with AS, who are primary candidates for TAVR.

Additionally, it's noteworthy that the mean age of our study participants was relatively younger, around 70 years, potentially influenced by the higher proportion of bicuspid valve patients. This age distribution may not fully represent the typical TAVR candidate population, which is generally older. This factor should be considered when interpreting our findings, particularly their extrapolation to a more aging TAVR candidate population.

## 6. Conclusion

Our investigation reveals that systolic annular measurements are advisable for clinical evaluation in patients with AR. The systolic AnMin index, in conjunction with patient gender, may offer insights as potential predictors for variations in annular dimensions, though their role should be considered within the broader context of each patient's unique clinical profile.

## Data Availability

The original contributions presented in the study are included in the article/**Supplementary Material**, further inquiries can be directed to the corresponding author.

## References

[1] Vahanian A Beyersdorf F Praz F Milojevic M Baldus S Bauersachs J 2021 ESC/EACTS Guidelines for the management of valvular heart disease: Developed by the Task Force for the management of valvular heart disease of the European Society of Cardiology (ESC) and the European Association for Cardio-Thoracic Surgery (EACTS). European Heart Journal (2022) 43:561–632. 10.1093/eurheartj/ehab395 34453165

[2] Blanke P Weir-McCall JR Achenbach S Delgado V Hausleiter J Jilaihawi H Computed Tomography Imaging in the Context of Transcatheter Aortic Valve Implantation (TAVI)/Transcatheter Aortic Valve Replacement (TAVR). JACC: Cardiovascular Imaging (2019) 12:1–24. 10.1016/j.jcmg.2018.12.003 30621986

[3] Willson AB Webb JG Freeman M Wood DA Gurvitch R Thompson CR Computed tomography–based sizing recommendations for transcatheter aortic valve replacement with balloon-expandable valves: Comparison with transesophageal echocardiography and rationale for implementation in a prospective trial. Journal of Cardiovascular Computed Tomography (2012) 6:406–414. 10.1016/j.jcct.2012.10.002 23127390

[4] Alharbi AA Khan MZ Osman M Khan MU Munir MB Syed M Transcatheter Aortic Valve Replacement vs Surgical Replacement in Patients With Pure Aortic Insufficiency. Mayo Clinic Proceedings (2020) 95:2655–2664. 10.1016/j.mayocp.2020.07.030 33276838 PMC8404150

[5] Huded CP Allen KB Chhatriwalla AK. Counterpoint: challenges and limitations of transcatheter aortic valve implantation for aortic regurgitation. Heart. (2021) 107(24):1942–5. 10.1136/heartjnl-2020-318682 33863760

[6] Bhogal S Rogers T Aladin A Ben-Dor I Cohen JE Shults CC TAVR in 2023: Who Should Not Get It? The American Journal of Cardiology (2023) 193:1–18. 10.1016/j.amjcard.2023.01.040 36857839

[7] Reid A Blanke P Bax JJ Leipsic J. Multimodality imaging in valvular heart disease: how to use state-of-the-art technology in daily practice. Eur Heart J. (2021) 42(19):1912–25. 10.1093/eurheartj/ehaa768 33186469

[8] Zhu D Chen W Peng L Guo Y. Valve sizing for pure aortic regurgitation during transcatheter aortic valve replacement. JACC: Cardiovasc Interv. (2015) 8(2):372–3. 10.1016/j.jcin.2014.11.011 25700760

[9] Tomii D Okuno T Heg D Lanz J Praz F Stortecky S Basal Septal Hypertrophy and Procedural Outcome in Patients Undergoing Transcatheter Aortic Valve Replacement. JACC: Cardiovascular Interventions (2022) 15:1688–1690. 10.1016/j.jcin.2022.06.026 35907749

[10] Subramanyam P Legasto AC Al’Aref SJ Wong SC Truong QA. Potential impact of dynamic automated CT aortic annular measurements on outcomes for transcatheter aortic valve replacement sizing. Int J Cardiovasc Imaging. (2020) 36(11):2291–7. 10.1007/s10554-020-01928-z 32621038

[11] Steffen J Beckmann M Haum M Fischer J Andreae D Orban M Systolic or diastolic CT image acquisition for transcatheter aortic valve replacement – An outcome analysis. Journal of Cardiovascular Computed Tomography (2022) 16:423–430. 10.1016/j.jcct.2022.05.003 35637128

[12] Stortecky S Heg D Gloekler S Wenaweser P Windecker S Buellesfeld L. Accuracy and reproducibility of aortic annulus sizing using a dedicated three-dimensional computed tomography reconstruction tool in patients evaluated for transcatheter aortic valve replacement. EuroIntervention. (2014) 10(3):339–46. 10.4244/EIJV10I3A59 24273249

[13] Hamdan A Guetta V Konen E Goitein O Segev A Raanani E Deformation Dynamics and Mechanical Properties of the Aortic Annulus by 4-Dimensional Computed Tomography. Journal of the American College of Cardiology (2012) 59:119–127. 10.1016/j.jacc.2011.09.045 22222074

[14] Blanke P Russe M Leipsic J Reinöhl J Ebersberger U Suranyi P Conformational Pulsatile Changes of the Aortic Annulus. JACC: Cardiovascular Interventions (2012) 5:984–994. 10.1016/j.jcin.2012.05.014 22995887

[15] Queirós S Morais P Fehske W Papachristidis A Voigt J-U Fonseca JC Assessment of aortic valve tract dynamics using automatic tracking of 3D transesophageal echocardiographic images. Int J Cardiovasc Imaging (2019) 35:881–895. 10.1007/s10554-019-01532-w 30701439

[16] Qing PL Gang YZ Qun YJ Gang CZ Deng W Shao H. Dynamic assessment of aortic annulus in patients with aortic stenosis throughout cardiac cycle with dual-source computed tomography. Int J Cardiol. (2012) 158(2):304–7. 10.1016/j.ijcard.2012.04.112 22592026

[17] Khamooshian A Amador Y Hai T Jeganathan J Saraf M Mahmood E Dynamic Three-Dimensional Geometry of the Aortic Valve Apparatus–A Feasibility Study. Journal of Cardiothoracic and Vascular Anesthesia (2017) 31:1290–1300. 10.1053/j.jvca.2017.03.004 28800987

[18] De Heer LM Budde RPJ van Prehn J Mali WPThM Bartels LW Stella PR Pulsatile Distention of the Nondiseased and Stenotic Aortic Valve Annulus: Analysis With Electrocardiogram-Gated Computed Tomography. The Annals of Thoracic Surgery (2012) 93:516–522. 10.1016/j.athoracsur.2011.08.068 22137241

[19] de Heer LM Budde RPJ Mali W de Vos AM van Herwerden LA Kluin J. Aortic root dimension changes during systole and diastole: evaluation with ECG-gated multidetector row computed tomography. Int J Cardiovasc Imaging. (2011) 27(8):1195–204. 10.1007/s10554-011-9838-x 21359833 PMC3230759

[20] Boccalini S Bons LR van den Hoven AT van den Bosch AE Krestin GP Roos-Hesselink J Bicuspid aortic valve annulus: assessment of geometry and size changes during the cardiac cycle as measured with a standardized method to define the annular plane. Eur Radiol (2021) 31:8116–8129. 10.1007/s00330-021-07916-8 33895857 PMC8523432

[21] Sands MP Rittenhouse EA Mohri H Merendino KA. An anatomical comparison of human, pig, calf, and sheep aortic valves. Ann Thorac Surg. (1969) 8(5):407–14. 10.1016/S0003-4975(10)66071-7 5353458

[22] Sutton JP Ho SY Anderson RH. The forgotten interleaflet triangles: a review of the surgical anatomy of the aortic valve. Ann Thorac Surg. (1995) 59(2):419–27. 10.1016/0003-4975(94)00893-C 7847960

[23] Bertaso AG Wong DTL Liew GYH Cunnington MS Richardson JD Thomson VS Aortic annulus dimension assessment by computed tomography for transcatheter aortic valve implantation: differences between systole and diastole. Int J Cardiovasc Imaging (2012) 28:2091–2098. 10.1007/s10554-012-0018-4 22318541

[24] Murphy DT Blanke P Alaamri S Naoum C Rubinshtein R Pache G Dynamism of the aortic annulus: Effect of diastolic versus systolic CT annular measurements on device selection in transcatheter aortic valve replacement (TAVR). Journal of Cardiovascular Computed Tomography (2016) 10:37–43. 10.1016/j.jcct.2015.07.008 26239964

[25] Pontone G Andreini D Bartorelli AL Bertella E Mushtaq S Gripari P Comparison of Accuracy of Aortic Root Annulus Assessment With Cardiac Magnetic Resonance Versus Echocardiography and Multidetector Computed Tomography in Patients Referred for Transcatheter Aortic Valve Implantation. The American Journal of Cardiology (2013) 112:1790–1799. 10.1016/j.amjcard.2013.07.050 24045059

[26] Burman ED Keegan J Kilner PJ. Aortic root measurement by cardiovascular magnetic resonance: specification of planes and lines of measurement and corresponding normal values. Circ: Cardiovascular Imaging. (2008) 1(2):104–13. 10.1161/CIRCIMAGING.108.768911 19808527

[27] Holst T Petersen J Adam G Reichenspurner H Girdauskas E Tahir E. A novel technique to quantify aortic valve annulus deformation: a pilot study. J Card Surg. 2022;37(9):2734–7. 10.1111/jocs.16683 35690897

