# Supplementary material for: The myth of aortic valve annulus changes in aortic valve disease
Source: Front Cardiovasc Med. 2023 Dec 14;10:1302992. doi: 10.3389/fcvm.2023.1302992 (PMC10755897; doi:10.3389/fcvm.2023.1302992)
Supplement: Supplementary file 1 [file Table1.docx]

Supplement Table 1 Comparison of Echocardiographic Parameters in the Expanded Cohort, Subgrouped by Type of Aortic Valve Disease

|  | Patient with systolic enlargement | | Patient with diastolic enlargement | |
| --- | --- | --- | --- | --- |
|  | AS  (n = 45) | AR  (n = 38) | AS  (n = 13) | AR  (n = 9) |
| IVS, mm | 13 (12, 14) | 10.5 (9.25, 11.75) | 12 (11, 14) | 10 (9, 12) |
| LVEDD, mm | 52 (47, 55) | 61 (57, 64) | 52 (48, 58) | 63 (60 ,66) |

IVS: = interventricular septal; LVEDD = left ventricular end-diastolic dimension

Supplement Table 2 Definitions of Additional Clinical Parameters

| Calcification volume | Calcification volume was measured using a calcification threshold set at 850 HU. |
| --- | --- |
| Interventricular septum hypertrophy | Mid-septal thickness of 12mm or greater during diastole |
| Annulus calcification | The calcification occurring in the annulus |


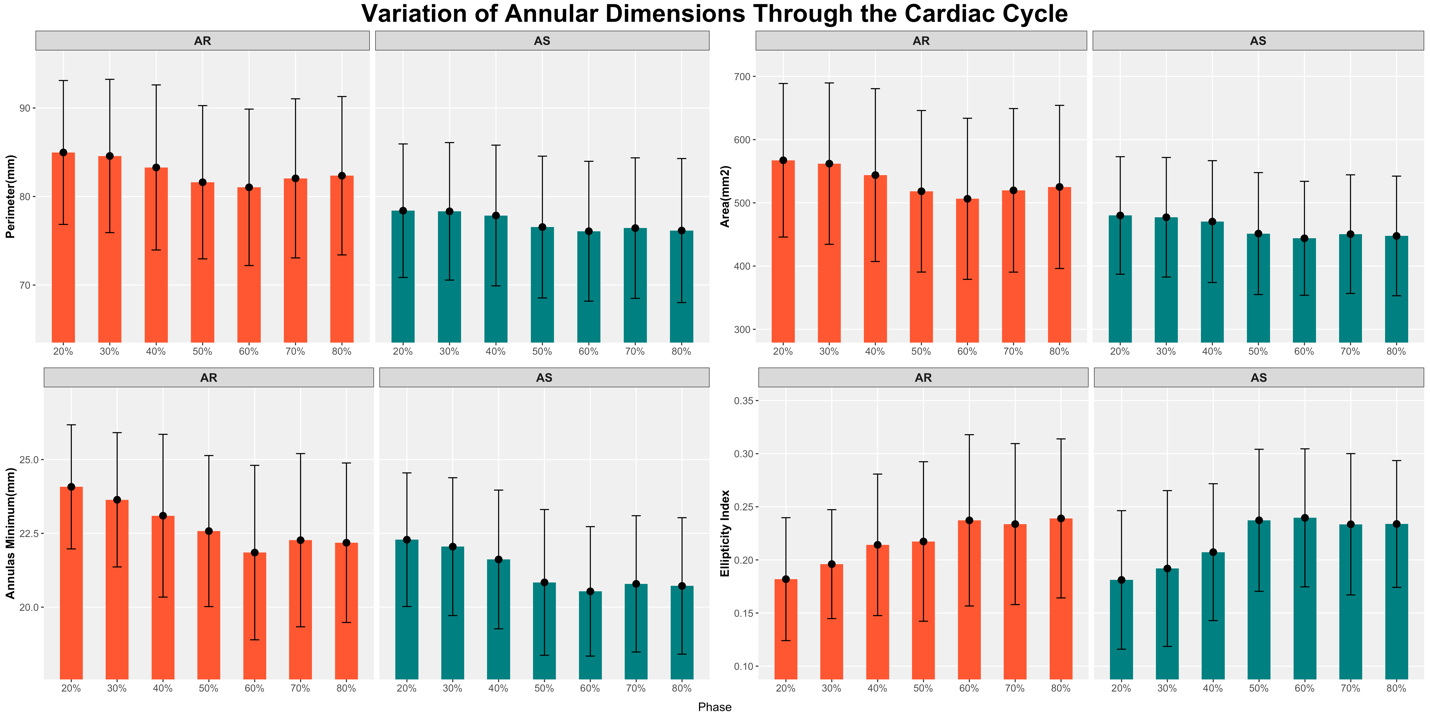


Supplement Figure 1 Variation of Annular Dimensions Throughout the Cardiac Cycle

AS = aortic stenosis; AR = aortic regurgitation


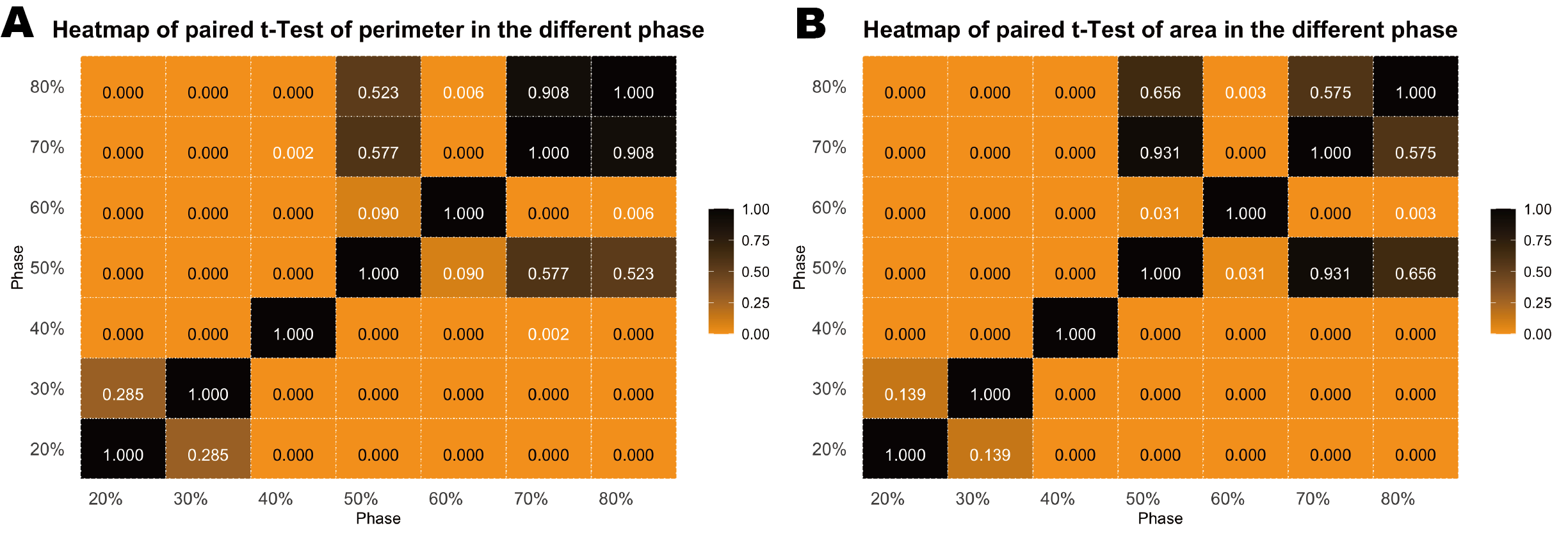


Supplement Figure 2 *P*-value matrices showing statistically significant differences between perimeter and area

Supplement Table 3 Criteria for valve sizing

|  | Evolut PRO+ | | | |
| --- | --- | --- | --- | --- |
| Size | 23mm | 26mm | 29mm | 34mm |
| AnPd | 18-19.9 | 20-22.9 | 23-25.9 | 26-30 |

AnPd = Perimeter-derived diameter for annulus
